# Voltage-Gated Sodium Channel Modulation by a New Spider Toxin Ssp1a Isolated From an Australian Theraphosid

**DOI:** 10.3389/fphar.2021.795455

**Published:** 2021-12-24

**Authors:** Yashad Dongol, Phil M. Choi, David T. Wilson, Norelle L. Daly, Fernanda C. Cardoso, Richard J. Lewis

**Affiliations:** ^1^ Centre for Pain Research, Institute for Molecular Bioscience, The University of Queensland, Brisbane, QLD, Australia; ^2^ Centre for Molecular Therapeutics, Australian Institute of Tropical Health and Medicine, James Cook University, Cairns, QLD, Australia

**Keywords:** ICK peptide, Ssp1a, spider toxin, venom peptide, voltage-gated sodium channel

## Abstract

Given the important role of voltage-gated sodium (Na_V_) channel-modulating spider toxins in elucidating the function, pharmacology, and mechanism of action of therapeutically relevant Na_V_ channels, we screened the venom from Australian theraphosid species against the human pain target hNa_V_1.7. Using assay-guided fractionation, we isolated a 33-residue inhibitor cystine knot (ICK) peptide (Ssp1a) belonging to the NaSpTx1 family. Recombinant Ssp1a (rSsp1a) inhibited neuronal hNa_V_ subtypes with a rank order of potency hNa_V_1.7 > 1.6 > 1.2 > 1.3 > 1.1. rSsp1a inhibited hNa_V_1.7, hNa_V_1.2 and hNa_V_1.3 without significantly altering the voltage-dependence of activation, inactivation, or delay in recovery from inactivation. However, rSsp1a demonstrated voltage-dependent inhibition at hNa_V_1.7 and rSsp1a-bound hNa_V_1.7 opened at extreme depolarizations, suggesting rSsp1a likely interacted with voltage-sensing domain II (VSD II) of hNa_V_1.7 to trap the channel in its resting state. Nuclear magnetic resonance spectroscopy revealed key structural features of Ssp1a, including an amphipathic surface with hydrophobic and charged patches shown by docking studies to comprise the interacting surface. This study provides the basis for future structure-function studies to guide the development of subtype selective inhibitors.

## Introduction

Voltage-gated sodium (Na_V_) channels are crucial for signalling in electrically excitable cells including nerve, heart and skeletal muscle (Ahern et al., 2016). Accordingly, Na_V_ channel dysfunction is associated with various diseases including pain (Cox et al., 2006; Vetter et al., 2017; Cardoso and Lewis, 2018; Bennett et al., 2019), migraine (Kahlig et al., 2008), epilepsy (Kaplan et al., 2016), multiple sclerosis (Waxman, 2006), cardiac arrythmia (Tarradas et al., 2013; Jeevaratnam et al., 2016) and myopathies (Cannon, 2010; Jurkat-Rott et al., 2010), supporting their therapeutic potential (Bagal et al., 2015; Kwong and Carr, 2015). Na_V_ channels comprise a single polypeptide chain arranged into four non-homologous domains, DI–DIV, which comes together to form the pore-forming *α*-subunit (de Lera Ruiz and Kraus, 2015). Humans have nine *α*-subunit isoforms hNa_V_1.1–1.9, each with distinct tissue localization, channel kinetics and physiological functions (Deuis et al., 2017b; Wu et al., 2018). Additional *β*-subunits associate with the *α*-subunit to regulate Na^+^ current kinetics and channel expression at the cell surface (Patino and Isom, 2010). These non-homologous domains and the channel pore provide key interaction sites for neurotoxins that either block the pore or modify channel gating (Stevens et al., 2011; Wu et al., 2018; Shen et al., 2019). Peptidic gating modifier toxins (GMTs) such as spider toxins preferentially target the extracellular face of domain II (neurotoxin receptor site 4) and domain IV (neurotoxin receptor site 3). In contrast, domains I and III are preferentially occupied by *β*-subunits, as observed in a recent cryo-EM structure of hNa_V_1.7-β1-β2 complexed with pore blockers and spider venom GMTs (Shen et al., 2019).

Spider venoms are a rich source of Na_V_ GMTs, with 12 families (NaSpTx1–12) defined based on intercysteine spacing and conserved amino acids (Klint et al., 2012). These spider toxins possess an inhibitor cystine knot (ICK) scaffold that provides structural, thermal, proteolytic and pH stability. By targeting voltage-sensing domains (VSD) II and IV, these spider toxins can affect channel activation or inactivation, respectively (Cardoso and Lewis, 2019; Dongol et al., 2019). Interestingly, some spider toxins appear to bind to both VSD II (Site 4) and VSD IV (Site 3), consistent with a more complex mode of action (Cardoso et al., 2017). Recently, the spider toxin Hm1a was used to investigate the role of Na_V_1.1 in chronic visceral pain and mechanical hypersensitivity (Osteen et al., 2016) but many more provide new molecular tools to help define the role and molecular pharmacology of Na_V_ channels. Increasingly, structure-function studies of these spider toxins are providing valuable new opportunities to rationally design potential drug leads with improved selectivity and potency (Xiao et al., 2010; Rong et al., 2011; Xiao et al., 2011; Cai et al., 2015; Wingerd et al., 2017; Xu et al., 2019; Jiang et al., 2021), especially NaSpTx1–3 family toxins ProTx-II (Flinspach et al., 2017), Pn3a (Deuis et al., 2017a; Mueller et al., 2019), HnTx-IV (Liu et al., 2014b), HwTx-IV (Liu et al., 2014a), and Ca2a (Zhang et al., 2018). In this study, we report the discovery of Ssp1a from an Australian theraphosid *Selenotypus* species and investigate its mode of action and selectivity across hNa_V_1.1–1.8. We conclude that Ssp1a is a gating modifier toxin that traps VSD II of Na_V_ channels in the closed (down) state without significantly altering the voltage-dependence of activation and inactivation.

## Materials and Methods

### Cell Culture

Cell culture reagents were from Life Technologies Corporation, CA, United States, unless otherwise stated. The human neuroblastoma cell line SHSY5Y was cultured in Roswell Park Memorial Institute (RPMI) medium supplemented with 15% fetal bovine serum (FBS) and 2 mM L-glutamine. Human embryonic kidney 293 (HEK293) cells stably expressing recombinant hNa_V_1.1–1.7 and the β1 auxiliary subunit (Scottish Biomedical Drug Discovery, Glasgow, United Kingdom) was cultured in Minimal Essential medium (MEM) (Sigma-Aldrich, MO, United States) supplemented with 10% v/v FBS-New Zealand origin (Assay Matrix), 2 mM L-glutamine and selection antibiotics as per manufacturer’s recommendation. Chinese hamster ovary (CHO) cells stably expressing hNa_V_1.8/β3 (ChanTest, Cleveland, United States) were cultured in MEM containing 10% v/v FBS and selection antibiotics as per manufacturer’s recommendation. All cells were maintained at 37°C in a humidified 5% CO_2_ incubator and sub-cultured every 3–4 days in a ratio of 1:5 using 0.25% trypsin/EDTA (Invitrogen).

### hNa_V_1.7 Screening

The crude venom of a female *Selenotypus* species was screened against hNa_V_1.7 in SH-SY5Y cells using a Fluorescent Imaging Plate Reader (FLIPR^Tetra^, Molecular Devices, CA, United States) as previously described (Cardoso et al., 2015; Cardoso et al., 2021). Briefly, SH-SY5Y cells were plated at 40,000 cells per well in 384-well flat clear-bottom black plates (Corning, NY, United States) and cultured at 37°C in a humidified 5% CO_2_ incubator for 48 h before commencing assays. Cells were loaded with 20 μL per well of Calcium 4 dye (Molecular Devices) reconstituted in assay buffer containing (in mM) 140 NaCl, 11.5 glucose, 5.9 KCl, 1.4 MgCl_2_, 1.2 NaH_2_PO_4_, 5 NaHCO_3_, 1.8 CaCl_2_, and 10 HEPES pH 7.4 and incubated for 30 min at 37°C in a humidified 5% CO_2_ incubator. Fluorescence responses were excited at 470–495 nm and emission recorded at 515–575 nm for 10 s to set the baseline, 600 s after addition of 250, 25 or 2.5 μg/ml venom, and for a further 300 s after addition of 4 μM veratridine and 30 nM scorpion toxin OD1.

### Venom Peptide Purification

Venom obtained from a female *Selenotypus* species (1 mg) was dissolved in 100 μL Milli-Q water containing 0.05% trifluoroacetic acid (TFA) (Auspep, VIC, Australia) and 5% acetonitrile (ACN) (Sigma-Aldrich, MO, United States). The sample was then centrifuged at 20,000 × *g* for 10 min to remove particulates. Venom was fractionated *via* reversed-phase high performance liquid chromatography (RP-HPLC) using a C18 column (Vydac 4.6 mm × 250 mm, 5 μm, Grace Discovery Sciences, United States) with a gradient of solvent B (90% ACN, 0.045% TFA in MilliQ water) in solvent A (0.05% TFA in MilliQ water). Fractionation started with isocratic elution at 5% solvent B for 5 min, followed by a gradient of 5–20% B from 5 to 10 min then a gradient of 20–40% solvent B over 40 min. The flow rate throughout was 0.7 ml/min, and 0.7 ml fractions were collected and lyophilized before storage at −20°C. The activity of each fraction on hNa_V_1.7 expressed in SHSY5Y cells was measured using FLIPR. The peptide mass of the most potent fraction was determined using a Triple TOF 5600 LC/MS/MS mass spectrometer (SCIEX, Framingham, MA, United States) using a C18 column (Zorbax 2.1 mm × 100 mm, 1.8 μm, Agilent) with a gradient of solvent B (0.1% formic acid in ACN) in solvent A (0.1% formic acid in ACN) at 60°C and flow rate 0.2 ml/min. N-terminal sequencing was outsourced to the Australian Proteome Analysis Facility. Briefly, peptides were reduced using dithiothreitol (25 mM) and incubated at 56°C for 30 min. The samples were then alkylated using iodoacetamide (55 mM) at room temperature for 30 min. The samples were purified *via* RP-HPLC using a Zorbax 300SB-C18 column (3 × 150 mm) (Agilent, CA, United States). 90% of the collected sample was loaded onto a pre-cycled Biobrene-treated disc and the sample was subjected to 38–42 cycles of Edman degradation using an ABI 494 Procise Protein Sequencing System (Applied Biosystems).

### Recombinant Production of Ssp1a

Recombinant Ssp1a (rSsp1a) was expressed in *E. coli* using the protocols previously described (Klint et al., 2013; Cardoso et al., 2015). Briefly, GeneArt Gene Synthesis (Life Technologies) was used to design a pLicC vector comprising Ssp1a gene, MalE signal sequence, maltose binding protein (MBP) tag, His_6_ affinity tag and a tobacco etch virus (TEV) protease recognition and cleavage sequence including other features necessary for periplasmic expression of Ssp1a in *E. coli*. The plasmid was transformed into BL21 (λDE3) competent *E. coli* cells and cultured in Luria-Bertani (LB) medium at 37°C, 120 rpm until optical density at 600 nm (OD_600_) reached 0.8–1.0. The expression of Ssp1a was induced at 16°C with 500 μM IPTG (isopropyl *β*-d-1-thiogalactopyranoside) and 120 rpm overnight. Cells were harvested at 6,000 rpm for 10 min at 4°C. The pellet was resuspended in TN buffer (Tris 25 mM, NaCl 150 mM, pH 8.0) and lysed in a constant pressure cell disruptor at 25 kPa. The lysate was centrifuged at 15,000 rpm, 4°C for 45 min and the supernatant containing the fusion protein was collected. The lysate supernatant was applied to Ni-NTA resin (Hispur NiNTA, Thermo Scientific) prewashed and equilibrated with TN buffer to capture the His-tagged fusion protein in the resin followed by washing with TN buffer containing 15 mM imidazole. The fusion protein was eluted by TN buffer containing 500 mM imidazole. The eluate was then desalted and concentrated in TN buffer using an Amicon centrifuge filter (30 kDa cut-off, Millipore). The fusion protein was cleaved by employing TEV protease at a final concentration of 0.02 mg/ml. The reducing environment required for TEV protease was maintained by using redox pair of glutathione (0.6 mM/0.4 mM GSH/GSSG). The effect of incubation temperature and time on TEV protease activity was monitored *via* gel (SDS-PAGE) analysis to determine the optimum temperature and time for TEV cleavage. The mixture of fusion protein, TEV protease and glutathione redox pair was incubated overnight at room temperature with gentle shaking at 100 rpm. The post-cleavage sample was filtered through a centrifuge filter to isolate the recombinant peptide. The filtrate which contained the peptide was applied on to a C18 column (30Å, 5 μm, 4.6 × 250 mm, Vydac 218TP, Grace) equilibrated with 5% solvent B (90% ACN, 0.05% TFA in MilliQ water) for RP-HPLC purification. A RP-HPLC fractionation was performed on Agilent 1,100 series using following gradient profile of solvent B in solvent A (0.05% TFA in MilliQ water): 5% solvent B over 0–5 min, 5–10% solvent B over 5–10 min, 10–50% solvent B over 10–40 min, 50–80% solvent B over 40–45 min, wash at 80% solvent B over 45–50 min, 80–5% solvent B over 50–55 min and a final wash with 5% solvent B over 55–65 min at a flow rate of 1 ml/min. Peak fractions were collected, analysed for the mass and purity, lyophilized, quantitated, and stored at −20°C until use. Matrix-assisted laser desorption/ionization time-of-flight mass spectrometry (MALDI-TOF-MS) was used to verify the mass of the recombinant peptide. The lyophilized fractions were reconstituted in Milli-Q water, mixed well with CHCA (α-cyano-4-hydroxycinnamic acid, 7 mg/ml in 50% ACN) matrix in a 1:1 (v/v) ratio and spotted on the MALDI plate. After drying out at room temperature, the plate was processed on a SCIEX 5800 MALDI-TOF/TOF and the spectra and data of monoisotopic [M + H]⁺ ions for rSsp1a acquired in positive reflectron mode.

### Automated Whole Cell Patch Clamp Electrophysiology

The HEK293 cells stably expressing hNa_V_1.1–1.7/β1 and CHO cells stably expressing hNa_V_1.8/β3 were prepared following manufacturer’s guidelines. After 48 h of incubation to achieve ∼80% confluency, cells were detached using Detachin (Genlantis) and resuspended to 5 × 10^6^ cells/mL in serum free media (CHO-cell SFM (Life Technologies), 25 mM HEPES and 100 U/mL penicillin/streptomycin). As hNa_V_1.8 expression in CHO cells is tetracycline inducible, the cells were further cultured for 24 h at 27°C in the presence of tetracycline (1 μg/ml). The electrophysiology experiments were conducted using the automated whole-cell patch clamp technology (QPatch 16X; Sophion Bioscience A/S, Ballerup, Denmark) as previously described (Cardoso et al., 2015; Cardoso et al., 2017) using QPlates with single patch hole/well for hNa_V_1.1–1.7 and 10 patch hole/well for hNa_V_1.8. The extracellular solution comprised (in mM) 1 CaCl_2_, 1 MgCl_2_, 5 HEPES, 3 KCl, 140 NaCl and 20 TEA-Cl with pH adjusted to 7.3 with NaOH. The intracellular solution comprised (in mM) 140 CsF, 1 EGTA, 5 CsOH, 10 HEPES and 10 NaCl with pH adjusted to 7.3 with CsOH. The osmolarity of both solutions was adjusted to 320 mOsm with sucrose. Compounds were prepared in extracellular solution containing 0.1% bovine serum albumin (Sigma-Aldrich). For experiments to record the outward Na^+^ currents in larger depolarization, modified solutions were used. The modified extracellular solutions contained (in mM) 105 NaCl, 5 CsCl, 35 choline chloride, 2 KCl, 10 HEPES, 1 MgCl_2_, 1 CaCl_2_, and 20 TEA-Cl with pH adjusted to 7.3. Similarly, the modified intracellular solutions contained (in mM) 108 NaCl, 35 CsF, 1 EGTA, 2 KCl and 10 HEPES, with pH adjusted to 7.3. Data was filtered at 3–8 kHz and sampled at 25 kHz. The mean seal resistance was 658 MΩ (95% confidence interval: 557–759 MΩ) while series resistance (Rs) was maintained below 10 MΩ with a mean for the last measured series resistance of 7 MΩ (95% confidence interval: 6.7–7.5 MΩ) with no compensation except for current-voltage experiments on hNa_V_1.2 and hNa_V_1.7, where fast Rs was compensated by 70%.

To obtain potency estimates at hNa_V_1.1–1.7, cells were maintained at a holding potential −80 mV and Na^+^ currents were elicited by 20 ms voltage step to 0 mV from a −120 mV conditioning pulse applied for 200 ms. Increasing concentrations of the peptide were incubated with the cells at holding potential for 5 min for native Ssp1a and 2 min for rSsp1a before the voltage protocol was applied. For CHO cells expressing hNa_V_1.8, cells were maintained at a holding potential of −90 mV and Na^+^ currents were elicited by 50 ms voltage step to +10 mV from a −90 mV conditioning pulse applied for 150 ms. For voltage-dependent inhibition of hNa_V_1.7, an IC_70_ concentration of rSsp1a was used and Na^+^ currents elicited by stepping cells to 0 and 50 mV for 20 ms from a −120 mV conditioning pulse applied for 200 ms.

The voltage protocols to determine voltage-dependence of activation and fast inactivation were combined, with cells held at −90 mV for 150 ms followed by step pulses from −110 mV to +75 mV in 5 mV increments to elicit the Na^+^ currents and determine voltage-dependence of activation. Each step pulse was maintained for 500 ms followed by a 10 ms pulse of −20 mV to elicit the Na^+^ currents to determine the voltage-dependence of steady-state fast inactivation. The cells were returned to −90 mV at 6 s intervals. Conductance-voltage curves were obtained by calculating the conductance (G) at each voltage (V) using equation G = I/(V–V_rev_), where I, V and V_rev_ represent the current value, membrane potential and reversal potential, respectively. For on-rate experiments, cells were maintained at a holding potential −80 mV and Na^+^ currents elicited by 20 ms voltage steps to 0 mV from a −120 mV conditioning pulse applied for 200 ms at 10 s interval for 300 s after addition of rSsp1a at 1x, 3x and 10x its IC_50_ at each hNa_V_ subtype analysed. The time constants for current block (Tau, τ_on_) at three concentrations were used to determine the actual on-rate (k_on_) for rSsp1a at hNa_V_ subtypes tested. The τ_on_ were used to determine k_obs_, where k_obs_ = 1/τ_on_. The calculated k_obs_ were plotted against their corresponding concentration to obtain a linear curve-fit where the slope of the curve represented the actual k_on_ (Dowling and Charlton, 2006; Pierre, 2011). For off-rate experiments, cells expressing hNa_V_1.2, hNa_V_1.3 and hNa_V_1.7 were maintained at the holding potential −80 mV and Na^+^ currents elicited by 20 ms voltage steps to 0 mV from a −120 mV conditioning pulse applied for 60 ms. Off-rates were determined using ∼ IC_50_ values of rSsp1a (250 nM, 500 and 130 nM, respectively) incubated for 5 min and Na^+^ currents measured every 3 min for 30 min during rSsp1a washout. The off-rate (k_off_) and dissociation constant (K_d_) values at three hNa_V_ subtypes were calculated using k_off_ = 1/τ_off_ (s^−1^) and K_d_ = k_off_/k_on_ (nM). Recovery from fast inactivation was examined using a two-pulse protocol where cells were conditioned at −120 mV for 200 ms were depolarized to 0 mV for 50 ms to inactivate the channels, followed by a step to −120 mV of variable duration (1–130 ms) to promote recovery, and a 50 ms test pulse to 0 mV to assess the availability of channels. To assess the effect of larger depolarizations on rSsp1a-bound hNa_V_1.7, cells were maintained at a holding potential of −90 mV and Na^+^ currents were recorded from a series of step depolarizations from −60 mV to +160 mV at 10 mV increment for 50 ms.

### Nuclear Magnetic Resonance Structure Determination of rSsp1a

Lyophilized peptide (500–1,000 μg) was resuspended in 90% H_2_O:10%D_2_O. 2D ^1^H–^1^H TOCSY, ^1^H–^1^H NOESY, ^1^H–^1^H DQF-COSY, ^1^H-^15^N HSQC, and ^1^H-^13^C HSQC spectra were acquired at 290 K, 298 and 305 K using a 600 MHz AVANCE III NMR spectrometer (Bruker, Karlsruhe, Germany) equipped with a cryogenically cooled probe. All spectra were recorded with an interscan delay of 1 s. NOESY spectra were acquired with mixing times of 200–250 ms, and TOCSY spectra were acquired with isotropic mixing periods of 80 ms. Two-dimensional spectra were collected over 4,096 data points in the f2 dimension and 512 increments in the f1 dimension over a spectral width of 12 ppm. Standard Bruker pulse sequences were used with an excitation sculpting scheme for solvent suppression. NMR assignments were made using established protocols (Wüthrich, 1983), and the secondary shifts derived by subtracting the random coil αH shift from the experimental αH shifts (Wishart et al., 1995). The three-dimensional structure of rSsp1a was determined using CYANA based on an automated assignment protocol for the non-intra-residue NOESY cross-peaks (Güntert, 2004). Torsion-angle restraints from TALOS+ were used in the structure calculations. One-dimensional and TOCSY spectra were recorded at 290 K, 298 and 305 K and referenced to internal 4,4-dimethyl-4-silapentane-1-sulfonic acid (DSS). The amide protons assigned at the different temperatures were used to calculate temperature coefficients based on Cierpicki and Otlewski, 2001 (Cierpicki and Otlewski, 2001). Hydrogen bond restraints were inferred from the analysis of the temperature coefficients and preliminary structures. Residues with temperature coefficients more positive than −4.6 ppb/K indicate involvement in hydrogen bonds (Cierpicki and Otlewski, 2001), with restraints for eight hydrogen bonds included in the structure calculations based on analysis of preliminary structures. Final structures were visualized using MOLMOL (Koradi et al., 1996).

### Molecular Docking

The recently solved structure of Na_V_Ab/Na_V_1.7 VS2A chimera (Wisedchaisri et al., 2021) VSD II and our NMR structure of rSsp1a were uploaded in HADDOCK2.2 Easy interface (Van Zundert et al., 2016) with structure and restraint definitions. We defined the active residues (W5, F6, P11, Y20, K25, W28, R30, Y31, and L33) for rSsp1a based on homology to related NaSpTx1 spider toxins HwTx-IV (Minassian et al., 2013) and GpTx-1 (Murray et al., 2015), while active residues on hNa_V_1.7 DII were defined based on the previously published channel mutation data (Xiao et al., 2010; Xiao et al., 2011; Cai et al., 2015; Zeng et al., 2018; Xu et al., 2019). The docking results were displayed as a cluster of water-refined models which were downloaded and visualized using Pymol 2.4.1 (DeLano, 2002).

### Data Analysis

The experimental data were analysed using QPatch Assay software v5.6.4 and GraphPad Prism 7.0 days using four-parameter Hill equation [Y = Bottom + (Top–Bottom)/(1 + 10^∧^(Log IC_50_-X)*Hillslope)] to fit concentration response curves by non-linear regression analysis, Boltzmann function [I(V) = I_Vmin_ + (I_Vmax_–I_Vmin_)/(1.0 + exp (–(V–V_50_/V_slope_)] for voltage-dependence of activation and inactivation, plateau followed by one-phase decay for on-rate, simple linear regression for actual on-rate determination, exponential growth equation for off-rate, one-phase decay for recovery from inactivation, one-way ordinary ANOVA (multiple comparisons) and Student’s *t*-test. Data are presented as mean ± standard error of mean (SEM) with number of independent experiments stated and *p* < 0.05 is considered statistically significant.

## Results

### Isolation of Ssp1a Spider Venom Peptide

**FIGURE 1:**
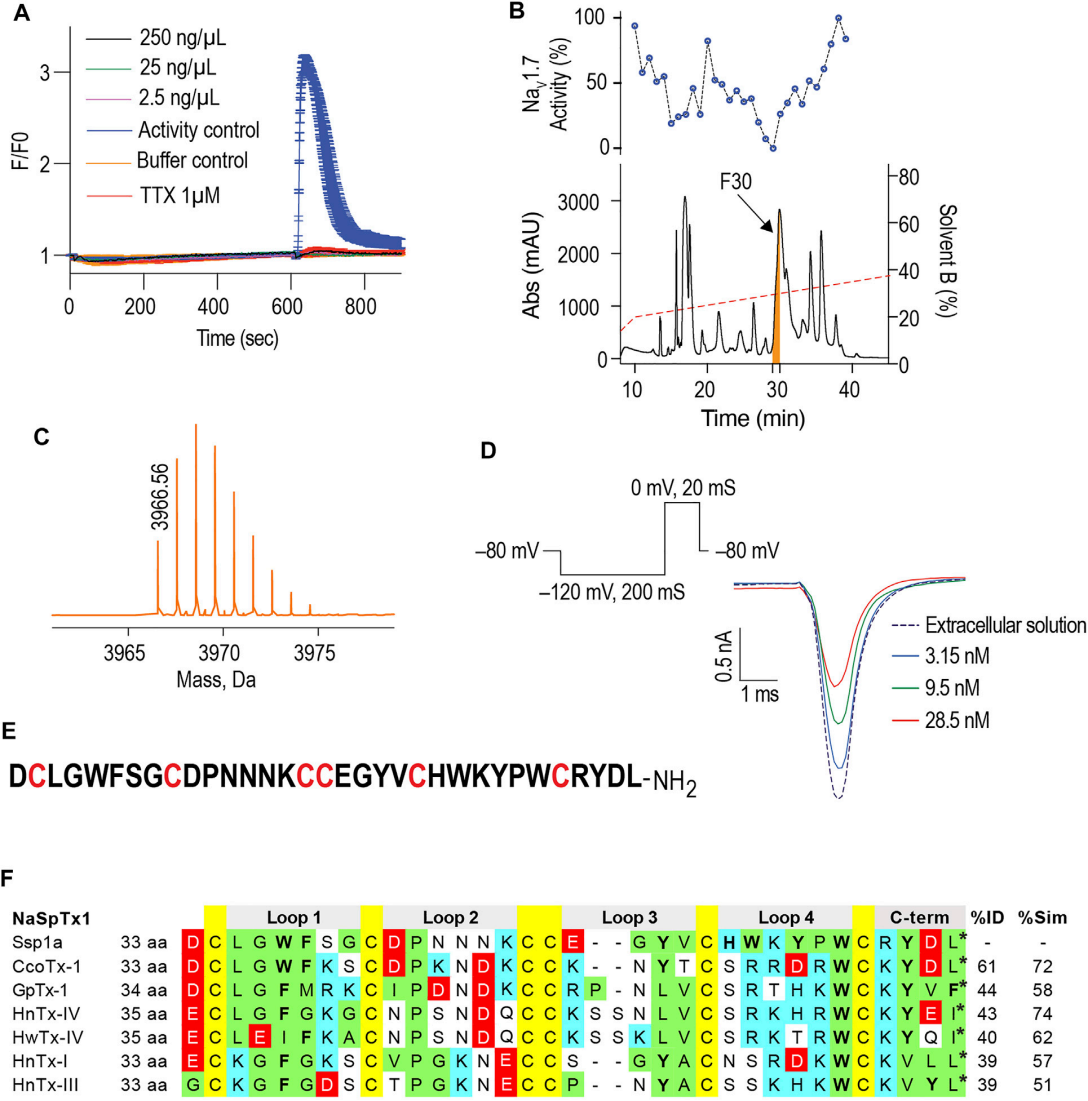
Isolation of the Australian tarantula peptide Ssp1a. **(A)** The crude venom of *Selenotypus* species was screened against hNa_V_1.7 at 250, 25 and 2.5 ng/ml measuring calcium influx into SH-SY5Y cells on a FLIPR^Tetra^. Tetrodotoxin (TTX) at 1 μM fully inhibited these veratridine (4 μM) + OD1 (30 nM) activated responses. **(B)** The crude venom was fractionated by RP-HPLC in C18 column using a gradient from 20 to 40% solvent B (dashed line) and 1-min fractions screened against hNa_V_1.7 to identify fraction 30 has the most prominent activity. **(C**) Reconstructed mass from Triple TOF LC/MS/MS spectra of fraction 30 revealed a single monoisotopic mass of 3,966.56 Da. **(D)** The inhibitory activity of fraction 30 against hNa_V_1.7 was confirmed by whole cell patch-clamp electrophysiology using HEK293 cells expressing hNa_V_1.7/β1 subunits on a QPatch 16X. Data are representative traces of *n* = 3. **(E)** N-terminal sequencing revealed the primary amino acids sequence of Ssp1a supporting the predicted monoisotopic mass of 3,966.60 Da with the C-terminal residue predicted to be leucine based on C-terminal tail homology. **(F)** Sequence alignment of Ssp1a with selected NaSpTx1 family toxins. Yellow highlights cysteines, green hydrophobic residues, cyan positively charged residues, red negatively charged residues, and bolded letters aromatic residues. The * indicates C-terminal amide.

hNa_V_1.7-specific calcium responses generated in SH-SY5Y cells using combination of veratridine and scorpion toxin OD1 provide a robust method to screen the hNa_V_1.7 blockers (Vetter et al., 2012). In these FLIPR^Tetra^ calcium assays, crude venom from the Australian Theraphosidae *Selenotypus* sp. inhibited hNa_V_1.7 at concentrations down to 2.5 ng/ml (Figure 1A). Screening the RP-HPLC fractions of *Selenotypus* sp. crude venom across hNa_V_1.7 revealed several fractions with inhibitory activity, especially fraction 30 (Figure 1B). MS analysis revealed fraction 30 contained a dominant peptide with a monoisotopic mass of 3,966.56 Da (Figure 1C). The Na_V_1.7 inhibitory activity of fraction 30 was confirmed using HEK293 cells recombinantly expressing hNa_V_1.7/β1 and automated whole cell patch-clamp electrophysiology in QPatch 16X (Figure 1D). The N-terminal sequencing revealed fraction 30 contained a 33-residue peptide with six cysteine residues typical for spider inhibitor cystine knot (ICK) peptides (Figure 1E). The C-terminal residue of Ssp1a was predicted to be either leucine or isoleucine from its monoisotopic mass, with leucine chosen considering C-terminal homology to related NaSpTx1 toxins. The sequence homology, amino acid residue number and intercysteine spacing categorised Ssp1a in the NaSpTx1 family (Klint et al., 2012), confirmed from its pairwise alignment with related NaSpTx1 family spider ICK peptides (Figure 1F).

### Recombinant Production and Structural Characterization of Ssp1a

**FIGURE 2:**
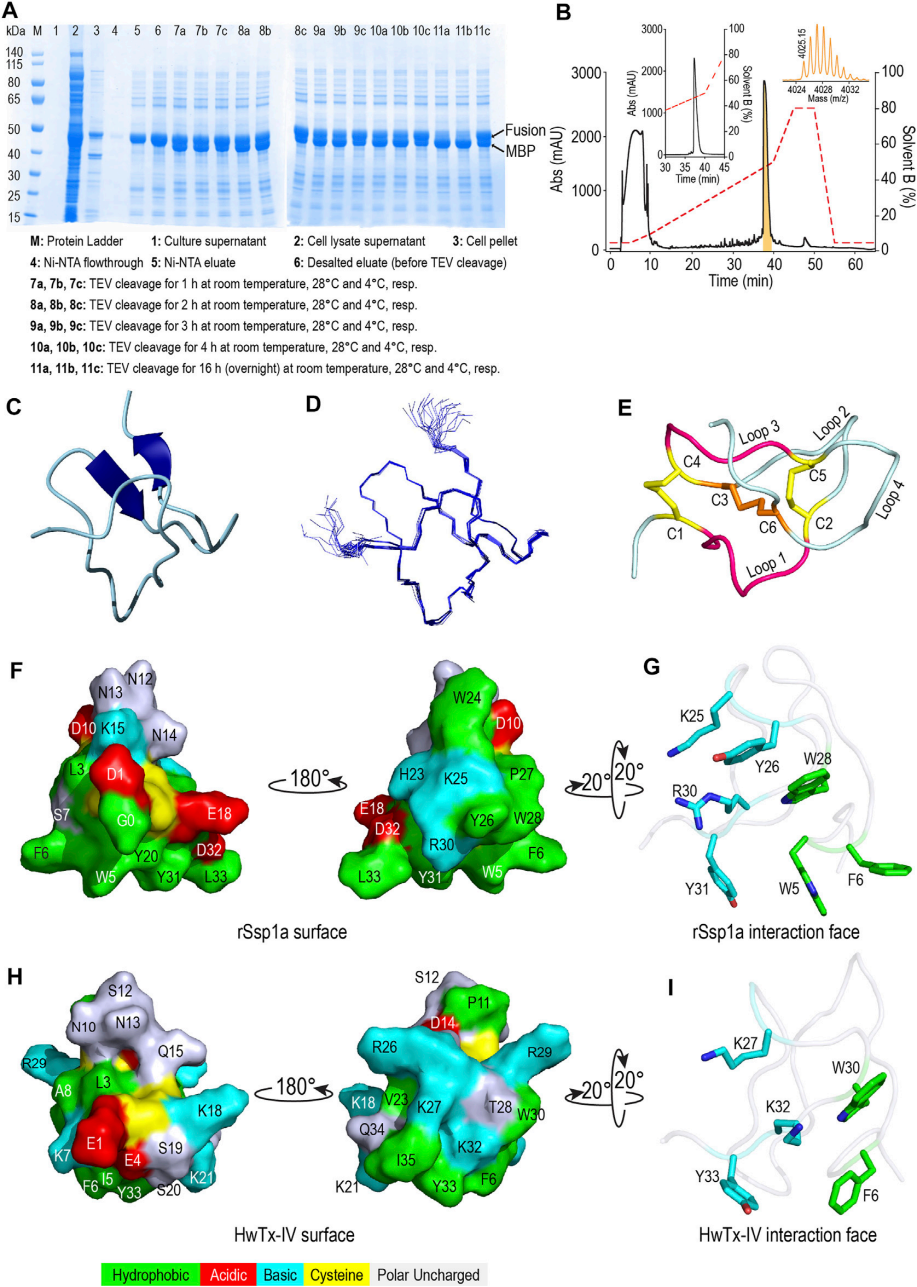
Recombinant production and structural characterization of rSsp1a. **(A)** SDS-PAGE analysis of the samples from various steps in recombinant expression of rSsp1a. **(B)** Analytical RP-HPLC (left inset) of rSsp1a obtained after semi-preparative RP-HPLC. The right inset shows the MALDI-TOF/TOF mass spectra of the purified rSsp1a. **(C,D)** NMR structure of rSsp1a showing a region of β-sheet connected by a β-hairpin at the C-terminus **(C)** and overlay of twenty lowest energy structures of rSsp1a **(D)**. **(E)** rSsp1a displaying an ICK motif. A ring structure made up of two disulfide bridges (yellow) and the intervening peptide backbone (pink) is penetrated by a third disulfide bridge (orange) to form a pseudo-knot. **(F,H)** Amphipathic surface profile of rSsp1a and HwTx-IV (PDB: 2M4X (Minassian et al., 2013)), respectively showing patches of hydrophobic residues (green) and charged residues (red: acidic residues and cyan: basic residues). **(G,I)** The interaction face of rSsp1a and HwTx-IV aligned to WCK/R motif characteristic to NaSpTx1 and 3 family toxins and showing important polar basic and polar hydrophobic residues (cyan) and hydrophobic residues (green).

Recombinant Ssp1a (rSsp1a) was expressed in the periplasm of BL21 (λDE3) *E. coli* strain as a fusion protein which was purified using Ni-NTA affinity chromatography followed by TEV protease cleavage to release the Gly-Ssp1a (rSsp1a). This N-terminal Gly residue is a remnant of TEV protease specific sequence (ENLYFQG) where TEV protease specifically cleaves between Q and G. The samples from each significant step of the expression protocol were analysed by SDS-PAGE to guide optimisation. The time and temperature kinetics of TEV cleavage was optimal at room temperature and 28°C for 16 h (Figure 2A), indicating the convenience to carry out the cleavage reaction at room temperature and also modified the previously used TEV cleavage temperature (30°C) in our lab at which 50% of the fusion protein was cleaved (Cardoso et al., 2015). Nickel affinity-purified rSsp1a eluted as a single peak at ∼ 50% solvent B from the C18 column, reflecting the relatively hydrophobic nature of this peptide (Figure 2B). The yield of rSsp1a after final RP-HPLC purification was 0.31 mg/L. The protonated monoisotopic mass ([M + H]^+^) of rSsp1a determined by MALDI-TOF/TOF was 4,025.99 m/z (Figure 2B, inset), consistent with the calculated monoisotopic mass (M, 4,024.61 Da) for native Ssp1a with an N-terminal Gly residue and C-terminal acid.

### Structure of rSsp1a

The structure of rSsp1a (PDB: 7SKC; BMRB: 30961) was determined using 2D NMR (Figure 2C), with an ensemble of 20 lowest-energy structures (Figure 2D) chosen to represent its structure (Supplementary Table S1). Ssp1a comprised a region of *β*-sheet connected by a *β*-hairpin at the C-terminus (Figure 2C) as the major element of secondary structure. This secondary structure, along with the disulfide connectivity (C1–C4, C2–C5, and C3–C6) and topology, constitute an ICK motif typical for spider ICK peptides targeting the Na_V_ channels (Klint et al., 2012). The ICK motif was formed by disulfide bridges C1–C4 and C2–C5 along with their intervening peptide backbone forming a ring through which the third disulfide bridge C3–C6 penetrates to form a pseudo-knot (Figure 2E). The hydropathicity/hydrophilicity analysis performed using Kyte and Doolitle hydropathy scale (Kyte and Doolittle, 1982; Gasteiger et al., 2005) revealed an amphipathic surface with uncharged hydrophobic amino acids contributing 26.5%, acidic and basic amino acids contributing 11.8% each, and polar uncharged (neutral) amino acids contributing 50% of the peptide (Figure 2F), like other Na_V_-targeting spider ICK peptides (Bosmans and Swartz, 2010), exemplified by HwTx-IV (Figure 2H). The surface arrangement of basic residues H23, K25 and R30 surrounded by hydrophobic residues W5, F6, Y26, W28 and Y31 (Figure 2G) on rSsp1a is predicted to represent the activity face of the peptide (Figure 2G), similarly to activity face defined for HwTx-IV (Figure 2I). π-Stacking between the aromatic rings of Y26 and W28 was also observed.

### rSsp1a Inhibits hNa_V_ Channel Subtypes

**FIGURE 3:**
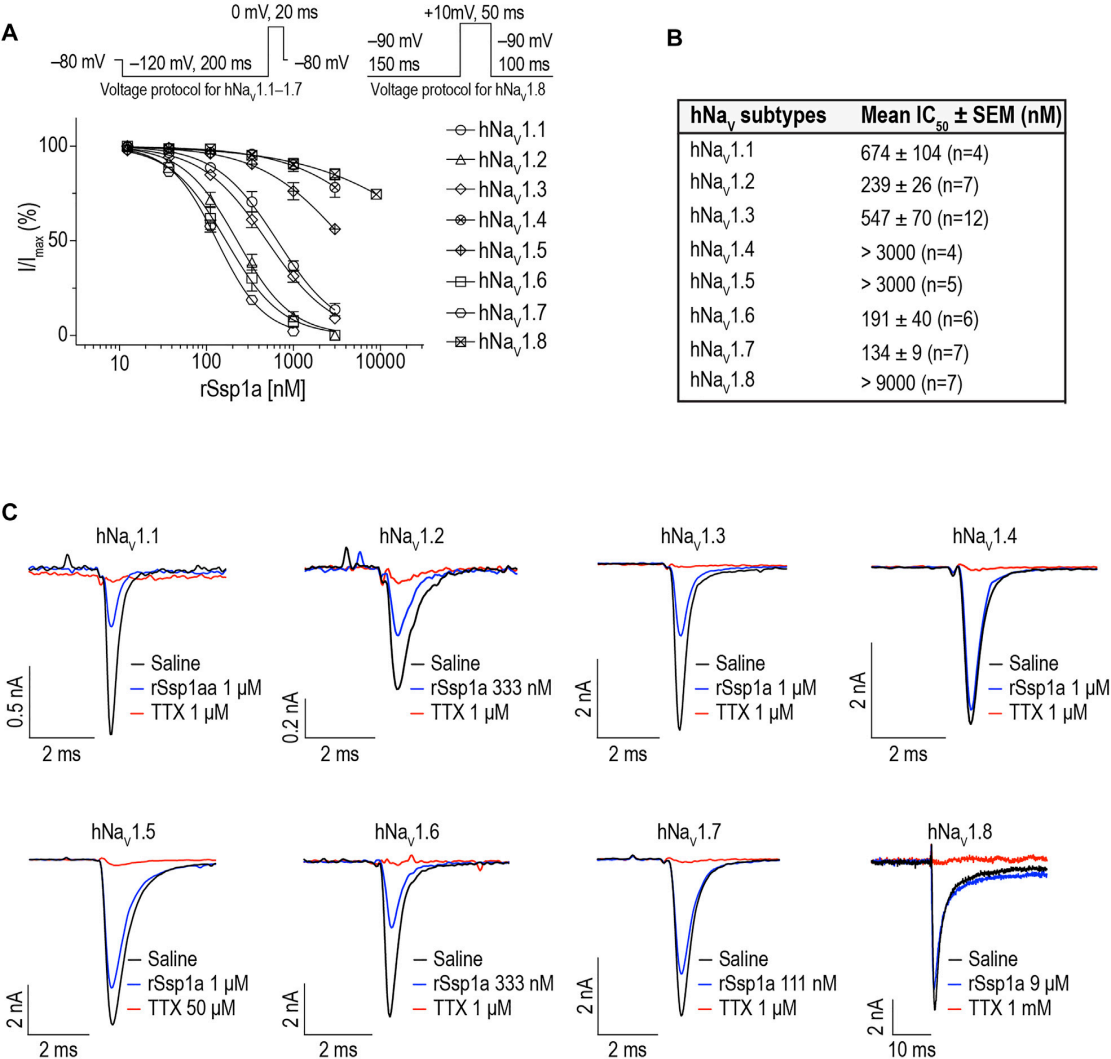
rSsp1a inhibition of hNa_V_ subtypes. **(A)** Dose-response profile of hNa_V_1.1–1.8 on automated-patch clamp electrophysiology platform (QPatch 16X). Voltage protocols used for dose-response assay on hNa_V_1.1–1.7 and hNa_V_1.8 are shown above the dose-response curve. **(B)** The mean IC_50_ ± SEM dose of rSsp1a observed at hNa_V_1.1–1.8 using electrophysiology assay. **(C)** Current traces of positive control (TTX, 1 μM for hNa_V_1.1–1.4, hNa_V_1.6 and hNa_V_1.7; 50 μM for hNa_V_1.5; and 1 mM for hNa_V_1.8), negative control (extracellular solution) and rSsp1a at varying concentration at hNa_V_1.1–1.8. CHO-cells expressing hNa_V_1.8 was used to obtain the dose-response curve on automated-patch clamp electrophysiology platform, otherwise HEK293 cells stably expressing hNa_V_1.1–1.7 were used to obtain the dose-response curve. Averaged data are shown as mean ± SEM (*n* = 4–12).

We determined the inhibitory potency of rSsp1a across hNa_V_1.1–1.7 stably expressed in HEK 293 cells and on hNa_V_1.8 stably expressed in CHO cells using automated whole-cell patch clamp electrophysiology (Figures 3A,B). rSsp1a inhibited hNa_V_ current at nanomolar concentrations, with a rank order of potency at TTX-S neuronal hNa_V_1.7 > 1.6 > 1.2 > 1.3 > 1.1. rSsp1a also blocked ∼60% hNa_V_1.5 current at 3 μM concentration, whereas it was weakly active at hNa_V_1.4 and hNa_V_1.8 at 3 and 9 μM, respectively. Figure 3C shows the representative current traces of hNa_V_1.1–1.8 channels after addition of negative control (extracellular solution), rSsp1a and the positive control TTX (1 μM for hNa_V_1.1–1.4, hNa_V_1.6 and hNa_V_1.7; 50 μM for hNa_V_1.5; and 1 mM for hNa_V_1.8).

### Effect of rSsp1a on Activation, Inactivation, and Recovery From Inactivation of hNa_V_1.2, hNa_V_1.3 and hNa_V_1.7

**FIGURE 4:**
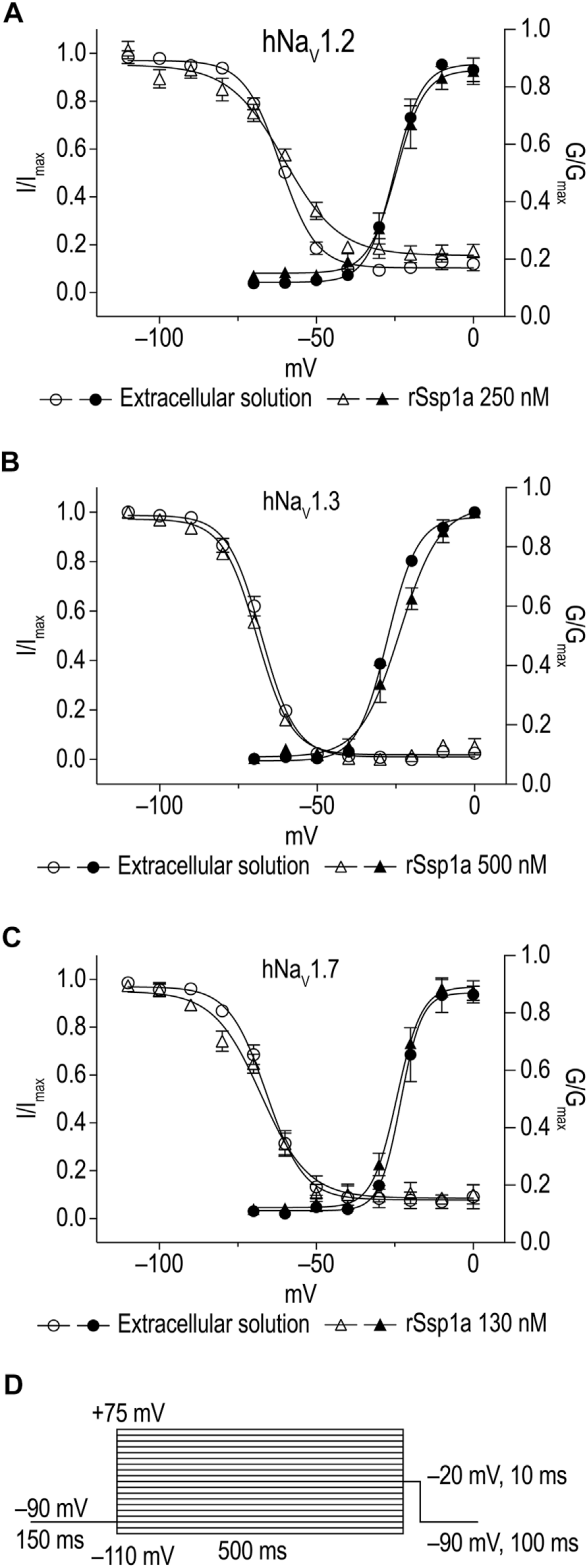
Current-voltage relationship of rSsp1a at hNa_V_1.2, hNa_V_1.3 and hNa_V_1.7. **(A–C)** show the voltage-dependence of activation and inactivation plotted as a function of G/G_max_ and I/I_max_. The corresponding I-V curves are shown in the Supplementary Figure S1. rSsp1a had little to no significant effects on the voltage-dependence of activation and steady-state inactivation. Data are represented as mean ± SEM (*n* = 3–4). **(D)** Voltage protocol used to determine the voltage-dependence of activation and inactivation.

**FIGURE 5:**
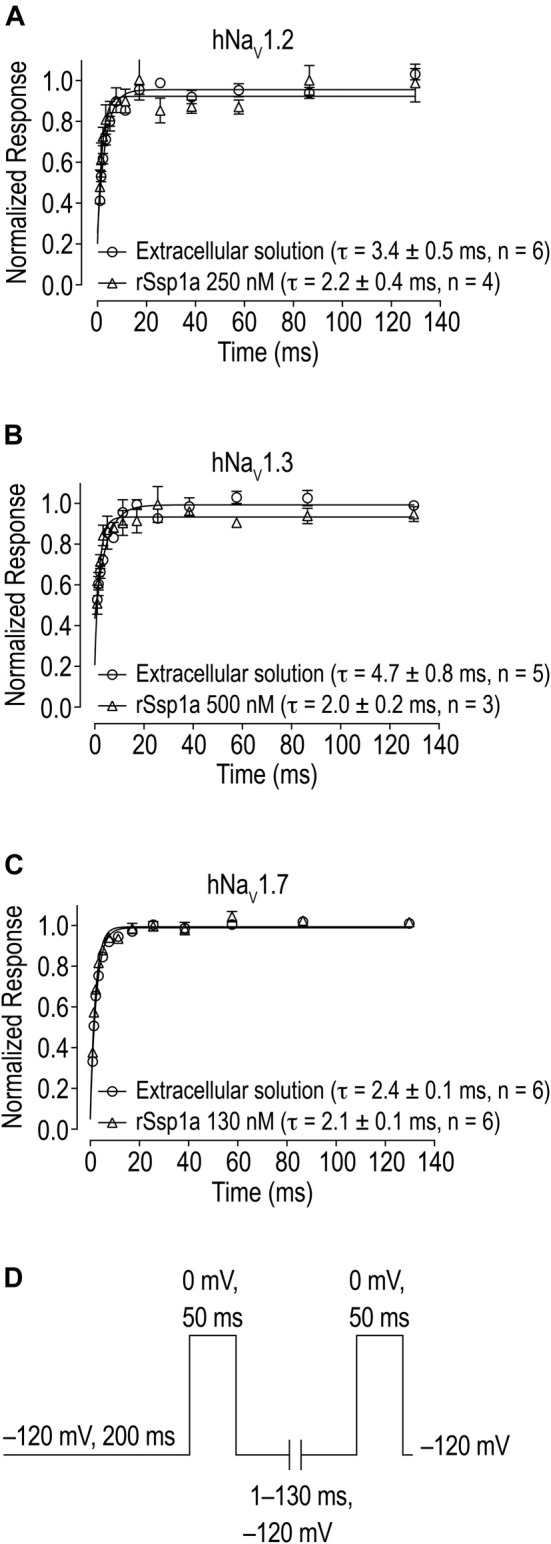
Effect of rSsp1a on the rate of recovery from inactivation. **(A–C)** rSsp1a does not delay the recovery rate from inactivation of hNa_V_1.2, hNa_V_1.3 and hNa_V_1.7, respectively. Data are shown as mean ± SEM, *n* = 3–6. **(D)** Voltage protocol used to determine the rate of recovery from inactivation.

The inhibition of therapeutically relevant hNa_V_1.2, hNa_V_1.3 and hNa_V_1.7 by rSsp1a at nanomolar potency supported our broader aim to extend the structure–function and rational design of NaSpTx beyond hNa_V_1.7 up to hNa_V_1.2 and hNa_V_1.3. To begin with, the basic pharmacology experiments to characterize the rSsp1a mode of action on channel gating were performed. As Na_V_-modulatory spider ICK peptides typically interact with VSD II associated with channel activation and/or VSD IV associated with channel inactivation (Dongol et al., 2019), rSsp1a effect on channel activation and inactivation was evaluated. At sub-saturating concentrations, rSsp1a had little or no significant effect on the voltage-dependence of steady-state activation and inactivation or the reversal potential on hNa_V_1.2, hNa_V_1.3 and hNa_V_1.7 (Table 1; Figures 4A–D). There was also no significant effect on the slope factor for voltage-dependence of activation at hNa_V_1.2 and hNa_V_1.7; however, at hNa_V_1.3 rSsp1a decreased the slope (*k* = 5.8 ± 0.4 mV) compared to the control (*k* = 4 ± 0.2 mV). On the other hand, for voltage-dependence of inactivation, the slope factor at hNa_V_1.3 remained unchanged whereas the steepness decreased by 3.5 mV for hNa_V_1.2 (−6.4 ± 0.5 mV, control Vs −9.9 ± 1.2 mV, 250 nM rSsp1a) and by 2.5 mV for hNa_V_1.7 (−9 ± 2.4 mV, control vs. −11.5 ± 2.2, 130 nM rSsp1a). rSsp1a also had no significant effect on recovery from fast inactivation at hNa_V_1.7 (τ value: control, 2.4 ± 0.1 ms vs. 130 nM rSsp1a, 2.1 ± 0.1 ms, *n* = 6) but accelerated recovery from fast inactivation at the hNa_V_1.2 by 1.2 mS (τ value: control, 3.4 ± 0.5 ms vs. 250 nM rSsp1a, 2.2 ± 0.4 ms, *n* = 4–6) and at hNa_V_1.3 by 2.7 ms (τ value: control, 4.7 ± 0.8 ms vs. 250 nM rSsp1a, 2.0 ± 0.2 ms, *n* = 3–5) (Figures 5A–D).

**TABLE 1:** Effect of rSsp1a on voltage dependence of activation and steady-state inactivation. Data are shown as mean ± SEM (*n* = 3–4), with statistical significance determined using the Student’s *t*-test (unpaired).

		Activation (mean ± SEM)			Inactivation (mean ± SEM)		
		V_50_ (mV)	k (mV)	*n*	V_50_ (mV)	k (mV)	*n*
hNa_V_1.2	Extracellular solution	−24.5 ± 0.8	4.8 ± 0.3	4	−62.2 ± 0.5	−6.4 ± 0.5	4
	250 nM rSsp1a	−23 ± 2.1^a^	5.3 ± 1.7^a^	4	−61.8 ± 0.8^a^	−9.9 ± 1.2^b^	4
hNa_V_1.3	Extracellular solution	−28 ± 0.3	4 ± 0.2	3	−67.8 ± 0.8	−5.4 ± 0.4	3
	500 nM rSsp1a	−24 ± 1.3^b^	5.8 ± 0.4^b^	3	−69.4 ± 0.4^a^	−5.6 ± 0.4^a^	3
hNa_V_1.7	Extracellular solution	−22.3 ± 2	4.4 ± 1.4	3	−64.3 ± 1.1	−9 ± 2.4	3
	130 nM rSsp1a	−24 ± 0.2^a^	4.9 ± 0.8^a^	3	−66.3 ± 1.2^a^	−11.5 ± 2.2^a^	3

a Statistically insignificant (*p* > 0.05) compared to control (extracellular solution).

b Statistically significant (*p* < 0.05) compared to control (extracellular solution).

### On-Rate and Off-Rate Effect of rSsp1a at hNa_V_1.2, hNa_V_1.3 and hNa_V_1.7

**FIGURE 6:**
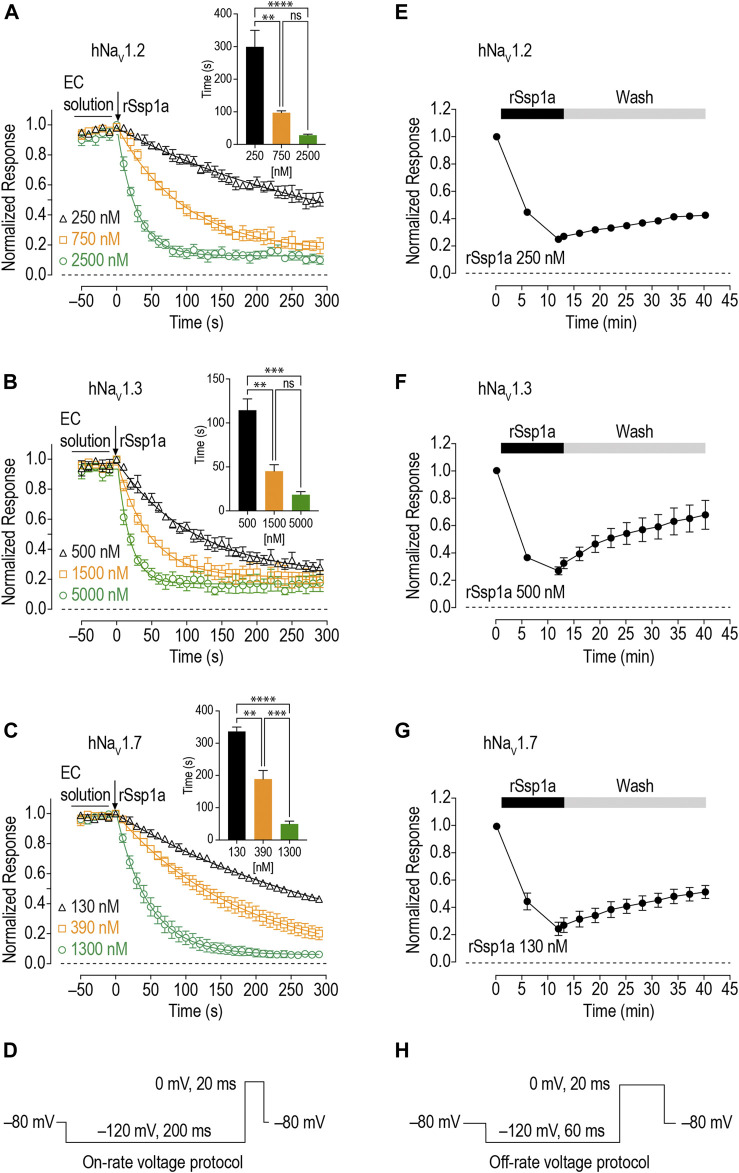
On-rate and off-rates for rSsp1a binding at hNa_V_1.2, hNa_V_1.3 and hNa_V_1.7. **(A–C)** On-rates for different concentrations of rSsp1a at hNa_V_1.2 (*n* = 4–6), hNa_V_1.3 (*n* = 3–4) and hNa_V_1.7 (*n* = 3–5), respectively. The Na^+^ currents were measured immediately after rSsp1a addition at 10 s interval for 300 s. The bar diagram for each plot compares the statistical significance of on-rate time constant between the rSsp1a concentrations. EC solution refers to extracellular solution. **(E–G)** Off-rate effects of 250 nM, 500 and 130 nM rSsp1a at hNa_V_1.2 (*n* = 6), hNa_V_1.3 (*n* = 6) and hNa_V_1.7 (*n* = 4), respectively. Cells were incubated for 5 min with rSsp1a at an ∼ IC_50_ concentrations and Na^+^ currents measured during washes with extracellullar solution every 3 min for 30 min. Data are shown as mean ± SEM. **, ***, and ****, and ns refer *p* values at < 0.01, < 0.001, < 0.0001, and > 0.05, respectively. **(D,H)** Voltage protocols used for on-rate and off-rate experiments.

As binding kinetics are an important consideration in drug development, we investigated the on- and off-rate for the inhibitory effects of rSsp1a at hNa_V_1.2, hNa_V_1.3 and hNa_V_1.7. rSsp1a on-rates were determined at an ∼ IC_50 (2 min)_ (250 nM for hNa_V_1.2, 500 nM for hNa_V_1.3, 130 nM for hNa_V_1.7), 3x IC_50_, and 10x IC_50_ concentrations (Figures 6A–D; Table 2). Off-rates at hNa_V_1.2, hNa_V_1.3 and hNa_V_1.7 revealed rSsp1a binding was slowly reversible and incomplete, with ∼25% of hNa_V_1.2, ∼50% of hNa_V_1.3 and ∼40% of hNa_V_1.7 channels recovering from block after a 30-min washout (Figures 6E–H). K_d_ was determined from the on-rate and reversible binding component at each hNa_V_ subtype, which revealed an ∼ 2-fold higher affinity of rSsp1a at hNa_V_1.7 (22.5 nM) and hNa_V_1.2 (19.5 nM) than at hNa_V_1.3 (37.0 nM) (Table 2).

**TABLE 2:** Association and dissociation kinetics of rSsp1a at hNa_V_1.2, hNa_V_1.3 and hNa_V_1.7. Data are shown as mean ± SEM (*n* = 3–6), while derived mean values were used to calculate K_d_. Statistical significance was determined using a one-way ANOVA with multiple comparisons.

	rSsp1a (nM)	τ_on_ (s)^a^	k_obs_ (s^−1^)	k_on_ (nM^−1^ s^−1^)	k_off_ (s^−1^)	K_d_ (nM)
hNaV1.2	250	299 ± 50	3.76 ± 0.64 × 10^−3b^	1.53 ± 0.18 × 10^−5^	2.98 ± 0.41 × 10^−4#^	19.5
	750	97 ± 6	1.05 ± 0.07 × 10^−2c^			
	2,500	28 ± 3	3.80 ± 0.45 × 10^−2d^			
hNaV1.3	500	115 ± 13	8.97 ± 1.07 × 10^−3e^	1.12 ± 0.21 × 10^−5^	4.13 ± 0.46 × 10^−4#^	37.0
	1,500	45 ± 7	2.36 ± 0.42 × 10^−2f^			
	5,000	18 ± 4	6.04 ± 1.04 × 10^−2g^			
hNaV1.7	130	336 ± 14	2.98 ± 0.13 × 10^−3h^	1.75 ± 0.29 × 10^−5^	3.93 ± 0.82 × 10^−4#^	22.5
	390	189 ± 27	5.58 ± 0.68 × 10^−3i^			
	1,300	50 ± 9	2.27 ± 0.35 × 10^−2j^			

k_obs_ = 1/τ_on_, where k_obs_ is observed k_on_; k_off_ = 1/τ_off_; K_d_ = k_off_/k_on_; k_on_ is derived by plotting k_obs_ at three concentrations against their respective concentration to obtain a linear curve-fit (y = mx + c), where the slope (m) represented the actual k_on_. ^a^ refer Figures 6A–C for corresponding *p* values; ^b and c, e and f, h and i, #^ not significantly different (*p* > 0.05); ^c and d^ significantly different (*p* < 0.001); ^b and d^ significantly different (*p* < 0.0001); ^f and g^ significantly different (*p* < 0.05); ^e and g, i and j, h and j^ significantly different (*p* < 0.01).

### rSsp1a is a Gating Modifier

**FIGURE 7:**
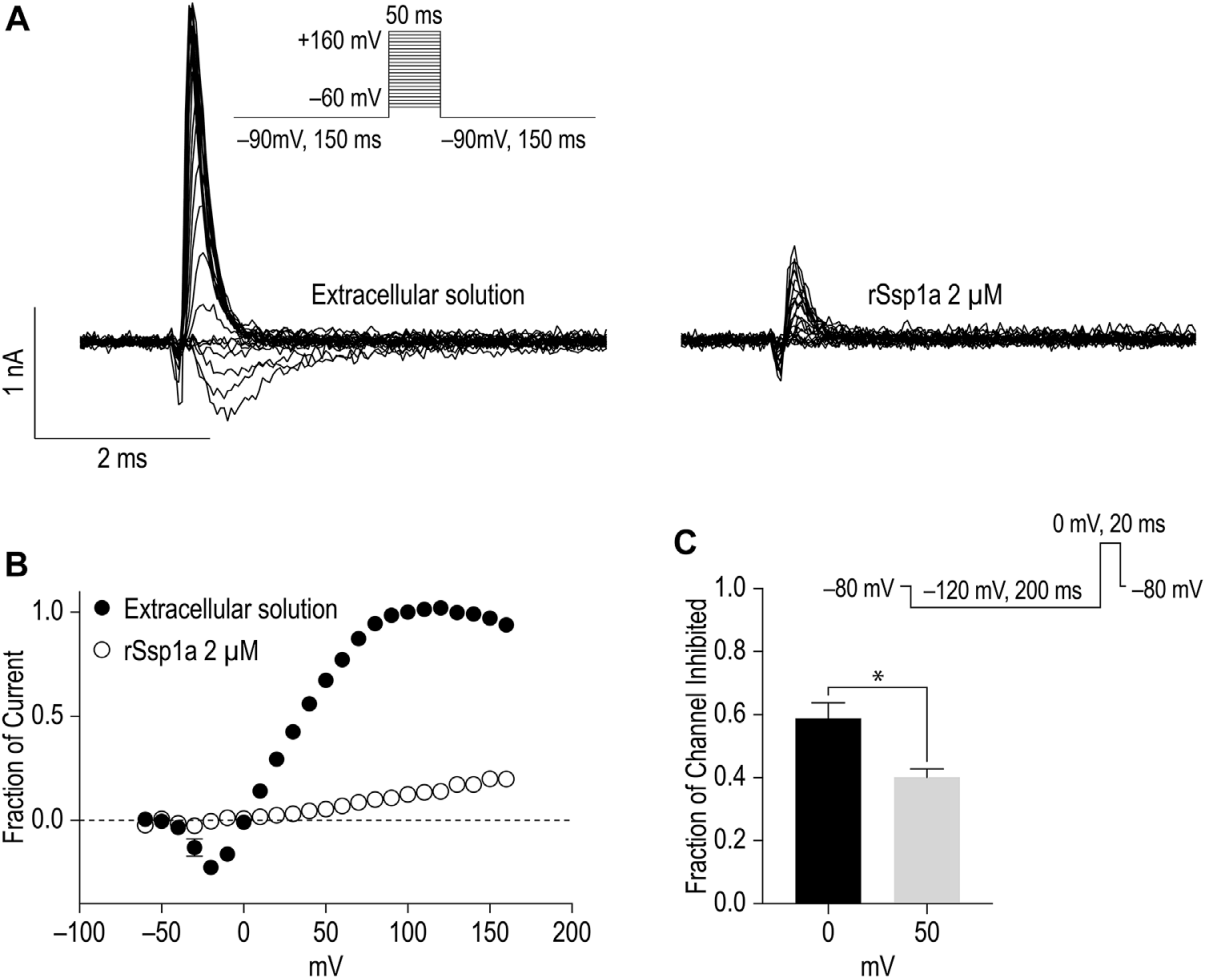
Voltage-dependent inhibition of hNa_V_1.7 by rSsp1a. **(A)** A current-voltage assay of hNa_V_1.7 at strong depolarizing potential ranging from −60 to +160 mV. The hNa_V_1.7 currents were recorded before (left) and after (right) application of toxin. **(B)** Current-voltage plot of peak current amplitude obtained before and after application of 2 μM rSsp1a (*n* = 5). Current amplitudes were normalized against the amplitude at −100 mV before rSsp1a application. **(C)** Voltage dependent inhibition of hNa_V_1.7 at 0 and 50 mV test pulse by 255 nM (IC_70_) rSsp1a (*n* = 4). The statistical significance was tested using Student’s *t*-test. Data are shown as mean ± SEM. *p < 0.05.

Gating modifiers can allow an outward current at high depolarizing potentials (Xiao et al., 2008; Liu et al., 2013; Cai et al., 2015). To investigate this phenomena, we depolarized cells from a holding potential of −90 mV to −60 mV to +160 mV for 50 ms using a modified extracellular and intracellular solutions to shift the reversal potential to 0 mV and enhance the amplitude of outward currents at strong depolarizing potentials as described by Xiao et al. (2008) (Figures 7A,B). Despite using a saturating concentration of rSsp1a (2 μM) to completely block the inward current at moderate depolarizing potentials, we observed a gradual increase in the outward current with increasing depolarizing potentials at ≥ 50 mV. The outward current observed for rSsp1a-bound hNa_V_1.7 at +160 mV was ∼21% compared to the current produced before rSsp1a application. This inferred that the rSsp1a-bound hNa_V_1.7 channels could be opened under strong depolarizing conditions. Further, rSsp1a (255 nM) inhibited ∼60% of hNa_V_1.7 channels at depolarisation to 0 mV compared to ∼40% at depolarisation to +50 mV (Figure 7C) confirming the toxin caused voltage-dependent inhibition of hNa_V_1.7.

### Molecular Docking of rSsp1a and hNa_V_1.7

**FIGURE 8:**
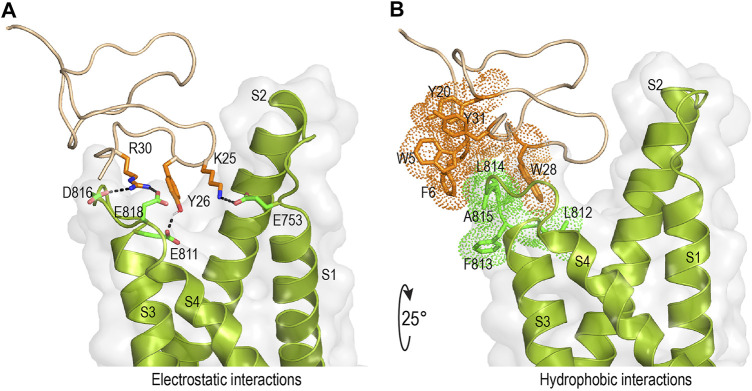
Molecular interaction of rSsp1a at hNa_V_1.7 VSD II. **(A,B)** Molecular docking results of rSsp1a at hNa_V_1.7 VSD II illustrating electrostatic and hydrophobic interactions. HADDOCK2.2 Easy interface (Van Zundert et al., 2016) was used to dock rSsp1a at recently solved resting structure of Na_V_Ab/Na_V_1.7-VS2A chimera (PDB 7K48) (Wisedchaisri et al., 2021) and the results were visualized using Pymol 2.4.1 (DeLano, 2002).

Mode of action studies suggest rSsp1a traps the VSD II of hNa_V_ channels in the resting conformation. Fortunately, the resting state of VSD II of Na_V_1.7 trapped by m3-HwTx-IV was recently solved (Wisedchaisri et al., 2021), allowing predictive docking of rSsp1a. Despite several studies suggesting membrane partitioning of spider toxin contributes to high-affinity toxin-channel interactions (Xu et al., 2019; Henriques et al., 2016; Milescu et al., 2007), the resolved structure of m3-HwTx-IV–Na_V_Ab/Na_V_1.7 VS2A we used for rSsp1a docking lacked a defined membrane-lipid environment and m3-HwTx-IV was allowed to interact directly with Na_V_Ab/Na_V_1.7 VS2A chimera (Wisedchaisri et al., 2021). HADDOCK2.2 was used to generate a molecular docking pose by defining rSsp1a active residues based on alanine scan data of closely related HwTx-IV (Minassian et al., 2013) and GpTx-1 (Murray et al., 2015) and published Na_V_1.7 channel mutational data (Xu et al., 2019; Xiao et al., 2010; Zeng et al., 2018; Cai et al., 2015; Xiao et al., 2011). The docking pose revealed that rSsp1a thrust itself to occupy the aqueous cleft formed in between S1–S2 and S3–S4 loop provisioning a strong salt bridge between K25–E753 (2.8 Å), R30–E818 (2.7 Å) and R30–D816 (2.7 Å), with further electrostatic interaction between Y26–E811 (2.0 Å) (Figure 8A). The hydrophobic stretch LFLA in the S3–S4 loop interacted with hydrophobic patch in rSsp1a comprising W5, F6, Y20, Y31, and W28 in a space filling model (Figure 8B). This binding mode is expected to further restrict the upward movement of S4 upon depolarization and thus trap VSD II in the resting state. Similar docking features were demonstrated by the closely related m3-HwTx-IV bound to the resting state VSD II in a recently captured cryo-EM structure, where the authors highlighted deep toxin penetration, stronger ionic interactions, and hydrophobic interactions at the S3–S4 loop that were important in locking the VSD II in the resting state (Wisedchaisri et al., 2021).

## Discussion

The advances in analytical and high-throughput technologies have overcome the hindrances of discovering and characterizing bioactive compounds from the smaller species such as spiders that produce miniscule amounts of venom (Escoubas et al., 2006; Pineda et al., 2014; Dongol et al., 2019). Pioneering work in the 1980s to elucidate the Na_V_-modulating effect of crude venom from *Phoneutria nigriventer* (Fontana and Vital-Brazil, 1985) and μ-agatoxins from *Agelenopsis aperta* (Adams et al., 1989) commenced the era of spider-venom toxin research. Initially these toxins served as a resource of novel ligands for ion channels and receptors to probe their structure–function (Escoubas et al., 2000; Stevens et al., 2011; Kalia et al., 2015; Wu et al., 2018) and later provided a novel library of potential drug leads (Saez et al., 2010; Pineda et al., 2014; Cardoso and Lewis, 2019; Saez and Herzig, 2019). Of the estimated >10 million bioactive peptides in the venom of spider species, < 0.01% have been described (Wilson, 2016), the majority of which targeted ion channels, importantly Na_V_s and Ca_v_s that are potential therapeutic targets (Smith et al., 2015). Building on this opportunity, we discovered and characterized a new Na_V_-modulatory toxin from the venom of an Australian theraphosid *Selenotypus* species with potential as a therapeutic lead.

### Isolation and Production of Ssp1a

Using hNa_V_1.7-guided fractionation of venom from a *Selenotypus* species, we isolated a new peptide, Ssp1a. Native Ssp1a is 33-residue long, C-terminal amidated venom peptide belonging to the NaSpTx1 family defined by conserved WCK/R motif, strongly conserved proline in loop 2, and intercysteine connectivity (C1–C4, C2–C5 and C3–C6) generating an anti-parallel β-sheet within a cystine knot (Klint et al., 2012). The closest homolog of Ssp1a with sequence identity 76% is a 32-residue μ-TRTX-Se1a isolated from *Selenocosmia effera* (Klint et al., 2015), which remains uncharacterized. Further, Ssp1a has a completely characterized homolog, HwTx-IV, that shares the same Na_V_-inhibitory mechanism and has 40% sequence identity with Ssp1a (Figure 1F). Thus, Ssp1a characterization might represent several closer uncharacterized homologs. A previously described bacterial expression system (Klint et al., 2013) efficiently produced the rSsp1a with a non-native N-terminal glycine TEV protease cleavage remnant and without C-terminal amidation. The periplasm expressed linear rSsp1a was folded under the redox environment provided by a GSH/GSSG redox pair. Nickel affinity chromatography and RP-HPLC separation techniques were used to purify rSsp1a as a single major product with the predicted mass (Figures 2A,B).

### Structure of rSsp1a

The 2D NMR analysis of this cysteine-rich rSsp1a confirmed that the peptide adopted a typical ICK fold (Figures 2C–E) which is the most prominent among the structural motifs, including disulfide-directed β-hairpin (DDH), Kunitz-type, colipase-like and helical arthropod-neuropeptide-derived (HAND), adopted by spider venom cysteine-rich peptides (Langenegger et al., 2019; Pineda et al., 2020). rSsp1a displayed an amphipathic surface comprising hydrophobic and charged patches (Figure 2F) often contributing to the interacting surface of ICK peptides (Figure 2H) (Lee and MacKinnon, 2004; Henriques et al., 2016; Lawrence et al., 2019). This surface typically includes functional residues (Y33 and K32) adjacent to hydrophobic residues (W30 and F6) that form the high affinity interface as shown for HwTx-IV (Figure 2I). Interestingly, rSsp1a surface displayed a cluster of K25, Y26 and R30 adjacent to a hydrophobic patch comprising W5, F6, Y26, Y31 and W28, that appears to facilitate charge-charge interactions (Figure 2G)**,** as supported by docking study discussed below.

### Pharmacology of rSsp1a

Whole-cell automated patch clamp electrophysiology revealed rSsp1a was inactive at hNa_V_1.4 and hNa_V_1.5, as well as at hNa_V_1.8 but dose-dependently inhibited neuronal hNa_V_1.7 > 1.6 > 1.2 > 1.3 > 1.1 (Figures 3A,B). The related HwTx-IV was similarly potent (IC_50_ 41–190 nM) at these five subtypes but had an altered rank order hNa_V_1.1 > 1.2 > 1.6 > 1.7 > 1.3 (Gonçalves et al., 2018). Interestingly, the rank order of potency of the Na_V_1.7 potency-optimized m3-HwTx-IV (hNa_V_1.7 > 1.6 > 1.3 > 1.1 > 1.2) was more similar to rSsp1a despite its enhanced potency (IC_50_ 3.3–11.9 nM) (Rahnama et al., 2017), while the most closely related CcoTx-1 had a different potency rank [hNa_V_1.2 (∼70 nM) > 1.1 (∼1,000 nM) > 1.7 (∼5,120 nM >> 1.3 and 1.6] in FLIPR assays (Sousa et al., 2017). These results exemplify that achieving improved subtype-selectivity can be challenging; however, lack of effect of these toxins on hNa_V_1.4 (muscle isoform) and hNa_V_1.5 (cardiac isoform) should simplify further development (Gonçalves et al., 2018).

The high potency of rSsp1a at hNa_V_1.7 indicates potential to inhibit pain signalling (Yang et al., 2004; Cox et al., 2006; Fertleman et al., 2006; Faber et al., 2012; Vetter et al., 2017). hNa_V_1.7 is a compelling target for pain therapeutics as silencing its activity completely caused pain insensitivity without any serious side effects except anosmia (Cox et al., 2006; Liu et al., 2014b; Tanaka et al., 2015; Deuis et al., 2017a; Flinspach et al., 2017; Zhang et al., 2018; Cardoso and Lewis, 2019; Mueller et al., 2019). rSsp1a potently inhibited hNa_V_1.6 (IC_50_ 191 nM) which plays a role in a variety of pain models, including oxaliplatin-induced cold allodynia (Sittl et al., 2012; Deuis et al., 2013), spinal nerve ligation induced mechanical pain (Xie et al., 2015), painful diabetic neuropathy (Ren et al., 2012) and trigeminal neuralgia (Tanaka et al., 2016), as well as in epilepsy (Blanchard et al., 2015; de Kovel et al., 2014; O’Brien and Meisler, 2013; Veeramah et al., 2012). However, the high expression of hNa_V_1.6 in peripheral motor neurons contributing to saltatory conduction of action potential may limit the utility of rSsp1a as a systemic analgesic lead (Rahnama et al., 2017; Gonçalves et al., 2018), although Na_V_1.6-selective blockers such as Xen901 (Bialer et al., 2018) and GS967 (Baker et al., 2018) have shown promise in treating epileptogenic disorders. The third most potently inhibited subtype hNa_V_1.2, localized in the central nervous system, is associated with epileptogenic channelopathies and might serve as an important therapeutic target for epilepsy-related disorders (Menezes et al., 2020). Similarly, rSsp1a also targeted hNa_V_1.3 suggesting potential to reverse pain phenotypes in animal models of neuropathic and inflammatory pain (Black et al., 1999; Kim et al., 2001; Hains et al., 2004; Hong et al., 2004; Garry et al., 2005; Lindia et al., 2005; Black et al., 2008; Chen et al., 2014; Tan et al., 2015; Xu et al., 2016) where it might be re-expressed in injured or affected sensory nerve (Waxman et al., 1994; Wang et al., 2011; Bennett et al., 2019). Lastly, the nanomolar inhibition of hNa_V_1.1 by rSsp1a suggest it may prove useful in reducing visceral pain and mechanical hypersensitivity (Osteen et al., 2016; Salvatierra et al., 2018).

Further pharmacological characterization was carried out on hNa_V_1.7, hNa_V_1.3 and hNa_V_1.2 that were most potently targeted by rSsp1a. rSsp1a reduced channel conductance with small effects on voltage-dependence of activation and inactivation at all three subtypes (Figure 4; Table 1), reminiscent of the inhibitory spider toxins HwTx-IV (Xiao et al., 2008; Xiao et al., 2010), HnTx-III (Liu et al., 2013) and HnTx-IV (Cai et al., 2015) from the NaSpTx1 family. Earlier studies suggesting HwTx-IV traps VSD II S4 in the closed (resting) state (Xiao et al., 2008; Xiao et al., 2010; Xiao et al., 2011) were confirmed by hNa_V_1.7 channel mutations (Cai et al., 2015; Liu et al., 2013; Xiao et al., 2008) and the recent cryo-EM structure analysis of the Na_V_Ab/Na_V_1.7-VS2A–m3-HwTx-IV complex (Wisedchaisri et al., 2021). Gating modifier spider ICK peptides can modulate the channel conductance by targeting neurotoxin Site 3 (domain IV) and Site 4 (domain II) in either the 1) down state of DII S4 (closed channel), 2) up state of DII S4 (open channel), and 3) down state of DIV S4 (open channel) (Dongol et al., 2019). Recently, the spider ICK peptide Tsp1a was reported to stabilize the inactivated state of the channel in the up state of DIV S4 (closed channel) (Jiang et al., 2021). Although rSsp1a’s effect on channel activation and inactivation at the three subtypes tested was independent of membrane potential, the extent of inhibition was voltage dependent (Figure 7C) and at saturating concentrations of rSsp1a, inward currents were fully inhibited at moderate depolarizations, while depolarizations above +50 mV partially restored current (Figures 7A,B). This phenomena is not observed with pore blockers, such as TTX (Xiao et al., 2008; Liu et al., 2013), indicating that rSsp1a is a gating modifier like HwTx-IV (Xiao et al., 2008), HnTx-IV (Cai et al., 2015), and HnTx-III (Liu et al., 2013) which trap VSD II in the closed state. Interestingly, rSsp1a did not alter the hNa_V_1.7 recovery from fast inactivation but slightly enhanced recovery from inactivation for hNa_V_1.2 and hNa_V_1.3 (Figure 5). On-rates were concentration-dependent, with rSsp1a binding to hNa_V_1.2 and hNa_V_1.7 at slower rate than to hNa_V_1.3 (Figures 6A–C; Table 2). Off-rates at hNa_V_1.2, hNa_V_1.7 and hNa_V_1.3 were slow and incomplete (Figures 6E–G) and the toxin had comparatively higher affinity for hNa_V_1.7 and hNa_V_1.2 than for hNa_V_1.3.

### Molecular Docking of rSsp1a at hNa_V_1.7

rSsp1a, HwTx-IV, HnTx-IV, and HnTx-III from the NaSpTx1 family share similar mechanisms of action and are expected to bind at overlapping sites on Na_V_ channels. Mutational (Xiao et al., 2008; Liu et al., 2013; Cai et al., 2015) and recent cryo-EM structures of Na_V_1.7 with bound HwTx-IV (Shen et al., 2019) or m3-HwTx-IV (Wisedchaisri et al., 2021) suggest rSsp1a might also bind to VSD II in the closed state. This was supported by docking studies that revealed rSsp1a bound to Na_V_1.7 similarly to m3-HwTx-IV to make important electrostatic and hydrophobic interactions with the S1–S2 and S3–S4 loop that would allow it to trap VSD II in the resting state (Wisedchaisri et al., 2021). Specifically, predicted functional residues K25 and R30 formed salt bridges with the acidic E753, D816 and E818, while Y26 H-bonded with E811 (Figure 8A). Docking suggests Y31 plays a lesser role in rSsp1a, whereas the equivalent Y33 in HwTx-IV is functionally critical (Figures 2G,I) (Minassian et al., 2013). The Y26 equivalent in HwTx-IV (T28) showed differential activity at hNa_V_ subtypes, as T28A-HwTx-IV had reduced potency (6-fold) at hNa_V_1.2 but similar potency at hNa_V_1.7 (Minassian et al., 2013). In contrast, the equivalent H27 in GpTx-1 was key to hNa_V_1.7 inhibition, with H27A-GpTx-1 showing a 10-fold potency reduction at this subtype (Murray et al., 2015). Comparison of the surfaces of rSsp1a and HwTx-IV (Figures 2F–I) revealed that the interacting face of these toxins comprises hydrophobic and basic residues distributed around the WCK/R motif. However, with 20% basic residues in HwTx-IV compared to 12% in rSsp1a, HwTx-IV contributes more basic residues to the binding face than rSsp1a, affecting charge distribution, hydrophobicity and toxin-membrane interaction (Henriques et al., 2016; Agwa et al., 2017; Lawrence et al., 2019). The low sequence identity of rSsp1a (40%) compared to HwTx-IV is expected to provide new opportunities to optimize its potency and/or hNa_V_ subtype selectivity.

Channel mutational (Xu et al., 2019; Xiao et al., 2010; Zeng et al., 2018; Cai et al., 2015; Xiao et al., 2011) and recent structural evidence (Xiao et al., 2011; Cai et al., 2015) have revealed that acidic residues in the VSD II S1–S2 loop (E753) and S3–S4 loop (E811, D816 and E818) provide the key toxin–channel interactions (Wisedchaisri et al., 2021). In addition to these charge interactions, hydrophobic interactions are also important in forming stable and high affinity toxin–channel complex. Accordingly, in the rSsp1a–hNa_V_1.7 VSD II docking, we observed the rSsp1a aromatic patch (W5, F6, Y20, W28, and Y31) contributed hydrophobic interactions with hNa_V_1.7 VSD II S3–S4 (L812, F813, L814, and A815) (Figure 8A). A similar set of interactions was also observed in the m3-HwTx-IV–Na_V_Ab/Na_V_1.7 VS2A complex (Wisedchaisri et al., 2021) where I5, F6, W30 and W33 interacted with the LFLA stretch in the S3–S4 loop to inhibit upward movement of voltage sensor S4 upon membrane depolarization.

## Concluding Remarks

We characterized a new spider toxin Ssp1a which potently inhibited neuronal Na_V_-channels with little effect on the voltage-dependence of activation and inactivation or delay in recovery from inactivation. These and docking data indicate Ssp1a traps VSD II of hNa_V_ channel in the resting state. Ssp1a is distantly related to well-characterized HwTx-IV (40% sequence identity) though each share the conserved WCK/R motif. Extending its pharmacological characterization to structure–function studies at hNa_V_1.2, hNa_V_1.3 and hNa_V_1.7 will help guide the development of potent and subtype selective inhibitors as well as provide new insights into the structure–function details to NaSpTx1 homologs more closely related to Ssp1a.

## Data Availability

The original contributions presented in the study are included in the article/Supplementary Material, further inquiries can be directed to the corresponding author. The dataset generated/analyzed for rSsp1a structure can be found in the Protein Data Bank (PDB code 7SKC; PDB DOI: 10.2210/pdb7skc/pdb) and Biological Magnetic Resonance Data Bank (BMRB Code: 30961).
