# Supplementary material for: Voltage-Gated Sodium Channel Modulation by a New Spider Toxin Ssp1a Isolated From an Australian Theraphosid
Source: Front Pharmacol. 2021 Dec 24;12:795455. doi: 10.3389/fphar.2021.795455 (PMC8740163; doi:10.3389/fphar.2021.795455)
Supplement: Supplementary file 1 [file DataSheet1.pdf]

Supplementary Table and Figure

Table S1. Structural statistics for the rSsp1a ensemble	
**Experimental restraints**	
Interproton distance restraints	395
Intraresidue	106
Sequential	126
Medium range (i-j < 5)	57
Long range (i-j ≥5)	106
Dihedral-angle restraints	32
Hydrogen bonds	16
**R.m.s. deviations from mean coordinate structure (Å)**	
Backbone atoms (residue 1-17)	0.76 ± 0.27
All heavy atoms (residue 1-17)	1.38 ± 0.22
**Ramachandran Statistics**	
% in most favoured region	79%
% in additionally allowed region	21%

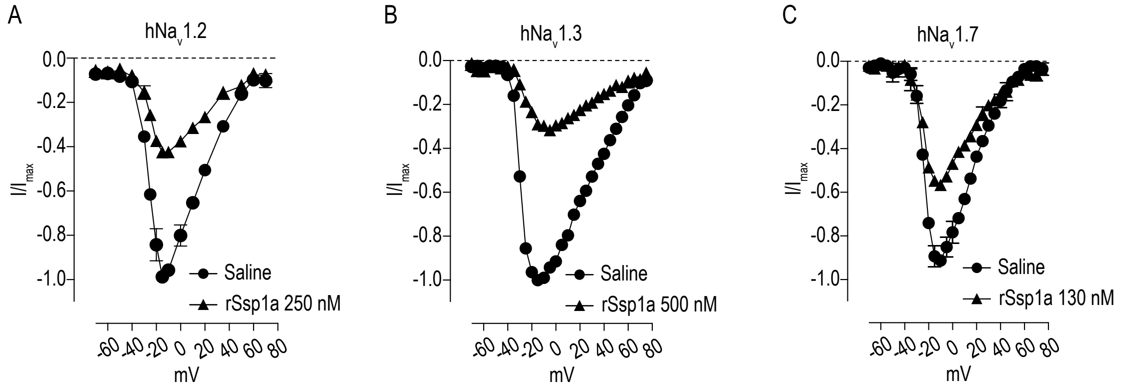

Figure S1. Effect of rSsp1a on current-voltage relationship of $hNa_V1.2$, $hNa_V1.4$ and $hNa_V1.7$. The ~IC50 rSsp1a reduced the peak current without significantly affecting the activation threshold, activation voltage and reversal potential. Each data point in the curve is mean ± SEM with n = 3–4.
